# The isovolumic relaxation to early rapid filling relation: kinematic model based prediction with in vivo validation

**DOI:** 10.1002/phy2.258

**Published:** 2014-03-20

**Authors:** Sina Mossahebi, Sándor J. Kovács

**Affiliations:** ^1^ Department of Physics College of Arts and Sciences Washington University in St. Louis St. Louis Missouri; ^2^ Cardiovascular Biophysics Laboratory Cardiovascular Division Department of Medicine Washington University in St. Louis St. Louis Missouri

**Keywords:** Diastolic function, echocardiography, hemodynamics, isovolumic relaxation, left ventricle

## Abstract

Although catheterization is the gold standard, Doppler echocardiography is the preferred diastolic function (DF) characterization method. The physiology of diastole requires continuity of left ventricular pressure (LVP)‐generating forces before and after mitral valve opening (MVO). Correlations between isovolumic relaxation (IVR) indexes such as tau (time‐constant of IVR) and noninvasive, Doppler E‐wave‐derived metrics, such as peak A‐V gradient or deceleration time (DT), have been established. However, what has been missing is the model‐predicted causal link that connects isovolumic relaxation (IVR) to suction‐initiated filling (E‐wave). The physiology requires that model‐predicted terminal force of IVR (*F*_*t*_ _IVR_) and model‐predicted initial force of early rapid filling (*F*_*i* E‐wave_) after MVO be correlated. For validation, simultaneous (conductance catheter) *P*‐*V* and E‐wave data from 20 subjects (mean age 57 years, 13 men) having normal LV ejection fraction (LVEF>50%) and a physiologic range of LV end‐diastolic pressure (LVEDP) were analyzed. For each cardiac cycle, the previously validated kinematic (Chung) model for isovolumic pressure decay and the Parametrized Diastolic Filling (PDF) kinematic model for the subsequent E‐wave provided *F*_*t*_ _IVR_ and *F*_*i* E‐wave_ respectively. For all 20 subjects (15 beats/subject, 308 beats), linear regression yielded *F*_*t*_ _IVR__ _= *α F*_*i* E‐wave_ + b (*R* = 0.80), where *α *= 1.62 and *b* = 1.32. We conclude that model‐based analysis of IVR and of the E‐wave elucidates DF mechanisms common to both. The observed in vivo relationship provides novel insight into diastole itself and the model‐based causal mechanistic relationship that couples IVR to early rapid filling.

## Introduction

Diastolic dysfunction (DD) is predictor of and a precursor to diastolic heart failure (DHF), a clinical syndrome that has reached epidemic proportions (Miller et al. 1986; Benjamin et al. 1992; Slotwiner et al. 1998; Zile and Brutsaert 2002; Kass et al. 2004; Maceira et al. 2006; Maeder and Kaye 2009). Critical to the management of this epidemic is the quantitative assessment of diastolic function (DF). DF determinants such as stiffness and relaxation, measured clinically, reflecting global chamber function have causal components at the cellular level. Physiologists and clinicians know that the LVP contour is smooth and continuous during the “isovolumic relaxation – mitral valve opening – early rapid filling” interval. The physiology of relaxation and filling requires continuity of left ventricular (LV) pressure generating forces before and after mitral valve opening (MVO). Correlations between invasive measures of isovolumic relaxation (IVR) such as tau (time‐constant of IVR) and E‐wave derived parameters, such as peak atrioventricular gradient or deceleration time (DT), have been established (Chung et al. 2006). However the known physiologic continuity that links these two phases has not been assessed in terms of the applicable kinematic models that individually allow computation of the model‐predicted force at the end of IVR and its relationship with the model‐predicted force at the beginning of suction initiated filling (E‐wave).

Quantification of diastolic dysfunction (DD) has remained a challenge without direct, invasive measurement. Doppler echocardiography has become the standard, and preferred method for quantitative DF assessment (Nishimura and Tajik 1997; Garcia et al. 1998; Khouri et al. 2004; Haney et al. 2005). In previous work, we have developed and validated novel, mechanism‐based DF indexes using a kinematic modeling approach, called the Parametrized Diastolic Filling (PDF) formalism (Kovács et al. 1987, 2000; Hall and Kovács 1994). The PDF formalism models the kinematics of suction‐initiated filling in analogy to the recoil from rest, of an equivalent damped oscillator. Model‐predicted velocity and clinical E‐wave contour velocity have shown superb agreement (Kovács et al. 2000). Using a clinically recorded E‐wave as input and suitable mathematical methods, unique chamber stiffness (*k*), viscoelasticity/relaxation (*c*) and load (*x*_*o*_) parameters are generated as output, thereby solving the ‘inverse problem of diastole’ (Kass et al. 2004). The three PDF parameters (*k, c, x*_*o*_) can be used to generate indexes with rigorous physiological analogues including the peak instantaneous pressure gradient (*kx*_*o*_), and the potential energy driving the recoil/suction process (1/2*kx*_*o*_^2^) (Bauman et al. 2004; Mossahebi et al. 2011). We have also previously derived and validated the ‘Chung model’, a kinematic model of isovolumic pressure decay (IVPD) applicable during IVR (Chung and Kovács 2008). The model accurately characterizes the wide range of physiologically observed IVPD contours when viewed as pressure phase plane (PPP) trajectories. It was shown that IVPD is governed by the interplay of inertial, stiffness and relaxation forces (Chung and Kovács 2008). Importantly, the Chung model is linear, it uses invasive high fidelity pressure contour as input and generates unique model parameters as output for each cardiac cycle. Furthermore, for the first time, the model unified the previous disparate characterizations of IVR in terms of *τ* or the logistic time‐constant *τ*_L_ by modeling the forces responsible and showing that linear (*τ*) and curved (*τ*_L_) fits to IVPD phases in the PPP are in fact parametric limits of a single unifying (Chung) model of IVR (Chung and Kovács 2008).

Thus, in this work, we employ the Chung model for IVPD and the PDF model for transmitral flow and compute the Chung model‐predicted expression for terminal force during IVR and PDF model‐predicted initial force initiating early rapid filling. Because the physiology is continuous, we hypothesize that the Chung‐model‐predicted terminal force of IVR (*F*_*t* IVR_) and the PDF model‐predicted initial force of early rapid filling (*F*_*i* E‐wave_) after MVO should be correlated.

## Materials and Methods

### Patient selection

Datasets from 20 patients (mean age 57 years, 13 men) were selected from our cardiovascular biophysics laboratory database of simultaneous echocardiography‐high fidelity hemodynamic (Millar conductance catheter) recordings (Lisauskas et al. 2001a; Chung and Kovács 2008). Subjects were referred by their personal physician for elective diagnostic cardiac catheterization to determine the possibility of coronary artery disease. Prior to data acquisition, subjects provided signed, IRB approved informed consent for participation in accordance with Washington University Human Research Protection Office (HRPO) criteria. The criteria for data selection from the database included: a range of LV end‐diastolic pressure (LVEDP) representative of a patient population encountered clinically, normal LVEF (>50%), normal sinus rhythm, clearly discernible E‐waves followed by a diastatic interval, and normal valvular function. Subject's inclusion in the study required the subject to have no pacemaker, be in normal sinus rhythm, have no evidence of valvular disease, and have no active ischemia. Among the 20 datasets, eight had end‐diastolic pressure (LVEDP) <15 mmHg, eight had 15 mmHg < LVEDP <20 mmHg, and four had LVEDP >20 mmHg. A total of 308 cardiac cycles of simultaneous echocardiographic‐high‐fidelity hemodynamic (conductance catheter) data were analyzed. The clinical descriptors of the 20 subjects and their hemodynamic and echocardiographic indexes are shown in Table 1.

**Table 1:** Clinical descriptors including hemodynamic and echocardiographic indexes

*N*	20
Age (years)	57 ± 11
Gender (male/female)	13 / 7
Heart Rate (bpm)	63 ± 6
Ejection Fraction (LVEF) (%)^a^	70 ± 7
LVEDP = MVOP (mmHg)	16 ± 4
LVEDV (mL)	127 ± 29
E/A	1.3 ± 0.2
PDF parameter *x*_*o*_ (cm)	9.6 ± 1.6
PDF parameter *k* (1/s^2^)	211 ± 44
PDF parameter *c* (1/s)	16.6 ± 4.1
Chung parameter *E*_k_ (1/s^2^)	1552 ± 763
Chung parameter *µ* (s)	0.013 ± 0.009

Data are presented as mean ± standard deviation.

LVEF, left ventricular ejection fraction; LVEDP, left ventricular end‐diastolic pressure; LVEDV, left ventricular end‐diastolic volume; MVOP, mitral valve opening pressure; E/A, ratio of E_peak_ and A_peak_.

a LVEF determined by ventriculography.

### Data acquisition

Our simultaneous high‐fidelity, *P*‐*V,* and echocardiographic transmitral flow data recording method has been previously detailed (Kovács et al. 1997, 2000; Lisauskas et al. 2001a; Chung et al. 2006; Mossahebi et al. 2011). Briefly, LV pressure was acquired using a micromanometric conductance catheter (SPC‐560, SPC‐562, or SSD‐1043, Millar Instruments, Houston, TX) at the commencement of elective cardiac catheterization, prior to the administration of iodinated contrast agents. Pressures signals were fed into the catheterization laboratory amplifier (Quinton Diagnostics, Bothell, WA, and General Electric) and simultaneously into the input ports of the physiological amplifier of the Doppler imaging system for synchronization (Philips iE33, Eindhoven, the Netherlands). Conductance catheterization signals were fed into a custom personal computer via a standard interface (Sigma‐5, CD Leycom). Although conductance volume data were recorded, analysis of the data was not necessary in this study.

### Doppler E‐wave analysis

For each subject, approximately 1–2 min of continuous transmitral flow data were recorded in the pulsed‐wave Doppler mode ([Link]). Echocardiographic data acquisition is performed in accordance with American Society of Echocardiography (Nagueh et al. 2009) criteria. Briefly, immediately before catheterization, patients were imaged in a supine position using a Philips iE33 system. Two dimensional images in apical two‐ and four‐chamber views were obtained. In accordance with convention, the apical four‐chamber view was used for Doppler E‐wave recording with the sample volume located at the leaflet tips. An average of 15 beats per subject was analyzed (308 cardiac cycles total for the 20 subjects). All E‐waves were analyzed using the Parametrized Diastolic Filling (PDF) formalism to yield E‐wave‐specific kinematic parameters (chamber viscoelasticity/relaxation parameter (*c*), stiffness parameter (*k*), load parameter (*x*_*o*_)) for each cardiac cycle (Kovács et al. 1987, 2001; Lisauskas et al. 2001b).

### Determination of terminal force of IVR using catheterization‐derived pressure data

Hemodynamics were recorded using high‐fidelity Millar LV pressure catheter for each beat. Kinematic model parameters of Chung model (*μ*,* E*_*k*_, and *P*_∞_), are extracted as previously described (Chung and Kovács 2008) for each individual beat by applying the Levenberg–Marquardt (LM) algorithm to the *P*(t) and d*P*(*t*)/d*t* data for isovolumic pressure decay (See [Link]).

**Figure 1:**
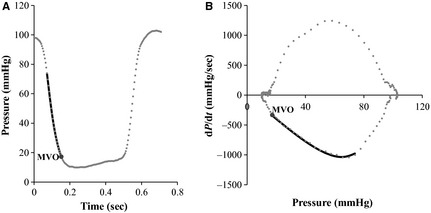
Chung model predicted isovolumic pressure decay up to mitral valve opening (MVO) employing elastic (*E*_*k*_) and relaxation (*μ*) parameters. (A) Raw data (gray dots) showing pressure versus time with model fit (solid black line) superimposed. (B) Chung model fit to same data in the pressure phase plane (d*P*/d*t* vs. *P*). Note ability of Chung model to fit curvilinear feature of IVR phase plane segment commencing at pressures greater than that at which negative d*P*/d*t* was greatest. See text for details.

Pressure is defined as force per unit area. When the Chung model (Fig. 1)‐predicted value of the pressure at MVO (P_MVO_) is multiplied by the effective (constant) mitral valve area (MVA) it provides the model‐predicted terminal force of IVR. For simplicity, effective MVA was considered as a constant (4 cm^2^). Therefore, the terminal force of IVR (*F*_*t* IVR_) is given by:

(1) $$F_{t\underset{}{}\mathrm{IVR}}=P_{\mathrm{MVO}}\cdot \text{MVA}$$

Because left atrial pressure is not routinely recorded during cardiac catheterization P_MVO_ very well approximated by LVEDP (Ishida et al. 1986; Murakami et al. 1986; Miki et al. 1991; Chung et al. 2004), and was the value used for terminating the Chung model‐predicted pressure in this study. Chung parameters which are computed per unit mass gives the force per unit mass (by setting *m* = 1), therefore, the unit of force becomes m/sec^2^.

### Determination of initial force of early rapid filling using echocardiographic data

PDF formalism solves the ‘inverse problem’ of diastole by providing three unique parameters, *k*,* c*, and *x*_*o*_, which specify each E‐wave contour (See [Link]). According to the PDF formalism, *k* is the stiffness parameter for early rapid filling. The initial displacement at MVO is given by *x*_*o*_ (cm). The force generated by a recoiling spring is the product of its stiffness and displacement. Therefore, the initial model‐predicted force of early rapid filling (*F*_*i* E‐wave_) applicable to E‐wave analysis is as follows:

(2) $$F_{i\underset{}{}E-\mathrm{wave}}=kx_{o}$$

As in previous work, PDF parameter values for *c*,* k*, and *x*_*o*_ are determined as output using the Levenberg–Marquardt algorithm using the E‐wave maximum velocity envelope as input via a custom Lab VIEW (National Instruments, Austin, TX) interface (Kovács et al. 2000; Dent et al. 2001; Chung et al. 2006; Zhang et al. 2010). As the PDF parameters (*c* and *k*) are computed per unit mass, the force computed from those parameters are computed per unit mass (by setting *m* = 1), therefore, the unit of force becomes m/sec^2^.

## Results

We analyzed 308 beats from 20 patients' datasets (~15 beats per person, 13 men). Demographics are shown in Table 1. Mean age was 57 years. Table 1 also lists mean heart rate, LVEF, LVEDV and LVEDP (=MVOP).

**Figure 2:**
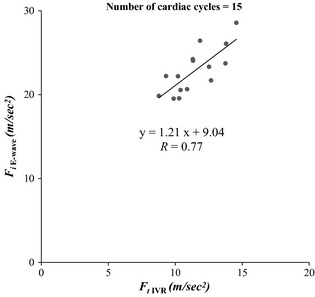
Initial force of early rapid filling (*F*_*i* E‐wave_) versus terminal force of isovolumic relaxation (*F*_*t*_ _IVR_) in one selected subject. Fifteen cardiac cycles were analyzed. Very good linear correlation was observed. See text for details.

When analyzed individually, a close linear relationship was found for the terminal force of IVR (*F*_*t* IVR_) and the initial force of early rapid filling (*F*_*i* E‐wave_) in accordance with the derivation (R > 0.71). Data from one subject are shown in Fig. 2. Individual linear regression for each dataset is shown in Table 2.

**Table 2:** Individual least mean square linear regression slopes for force relationship (*F*_*t IVR*_ and *F*_*i* E‐wave_) for 20 subjects

Subject	*F*_*i* E‐wave_ versus *F*_*t* IVR_	
	Linear fit slope	*R*
1	1.42	0.77
2	1.21	0.77
3	1.88	0.79
4	1.69	0.88
5	1.28	0.72
6	0.86	0.71
7	1.44	0.72
8	1.72	0.82
9	1.16	0.71
10	0.94	0.79
11	0.95	0.74
12	1.46	0.84
13	1.67	0.80
14	0.54	0.72
15	1.86	0.77
16	1.36	0.71
17	1.63	0.76
18	3.10	0.81
19	1.06	0.78
20	2.28	0.81

**Figure 3:**
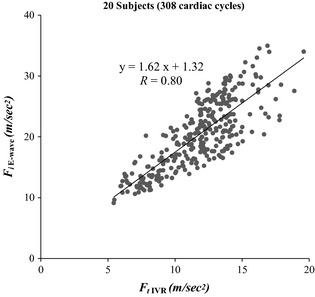
Initial force of early rapid filling (*F*_*i* E‐wave_) versus terminal force of isovolumic relaxation (*F*_*t*_ _IVR_) for the entire (20 normal) dataset consisting of 308 cardiac cycles. Very good linear correlation was observed. See text for details.

The relationship between *F*_*i* E‐wave_ and *F*_*t* IVR_ for the 20 datasets (308 beats) is shown in Fig. 3. It yielded a very good linear relationship *R* = 0.80.

## Discussion

Echocardiography is the preferred method of DF assessment. To provide a more complete set of causality‐ and mechanism‐based DF indexes, we used separate, independent kinematic models for IVR and early rapid filling phases of diastole. The advantage of these models is that they are linear, and therefore are ‘invertible’ generating numerically unique model parameters for each recorded IVR and subsequent E‐wave. In addition, both models use (Newton's) equations of motion (Eqs [A1] and [A3] in [Link]) and quantify the roles that inertial, resistive and restoring forces play in IVR and early rapid filling.

### Isovolumic relaxation models

Left ventricular isovolumic pressure decline is commonly characterized by the traditional relaxation indexes *τ* (the time‐constant of IVR) and *τ*_L_ (the logistic time‐constant). A more visually revealing and convenient way to characterize the IVR portion, and to assess model predicted fit to the data, is to plot it in the PPP, that is, a plot of the time derivative of pressure [d*P*/d*t*] versus time‐varying pressure [*P*(*t*)] (Fig. 1B). In the Weiss model, the rate of pressure decline as a function of time is assumed to be proportional to pressure itself, and the Weiss model generates –1/*τ* as the slope of the linear fit to the IVR segment, commencing below peak –d*P*/d*t* and terminating just above LVEDP in the PPP (Weiss et al. 1976). In the logistic model, the pressure decay during IVR is proportional to the square of the pressure, and it fits PPP trajectories having curvilinear, rather than linear IVR segments of (Matsubara et al. 1995). In other words, the Weiss model can only generate a straight line (linear) fit to the IVR portion of the PPP, whereas the logistic model can only generate a curvilinear fit to IVR contours in the PPP and no physiologic connection between *τ* and *τ*_L_ has been established and neither can be used to fit the PPP data before d*P*/d*t*_min_ (Chung and Kovács 2008).

In contrast, the Chung model provides excellent fits to the full range (linear or curved) IVR pressure decay contours encountered in the PPP and also fits PPP data before d*P*/d*t*_min_ (Chung and Kovács 2008). Importantly, the Chung model reveals that linear or curved IVR portions encountered in the PPP are mechanistically identical and correspond to parametric limits of a mechanistically single (Chung) model.

### Early rapid filling models

The PDF formalism models suction initiated early rapid filling (E‐wave). The relation between catheterization‐determined chamber stiffness (d*P*/d*V*) and *k*, and the viscoelasticity/ relaxation parameter *c* and the time‐constant of IVR *τ* have been previously established (Lakshminarayan K and SJ 1993; Hall and Kovács 1994; Kovács et al. 1997; Lisauskas et al. 2001a; Mossahebi et al. 2011).

### Expected correlation of isovolumic relaxation and early rapid filling measures

Correlations between invasive isovolumic relaxation (IVR) measures, such as tau (time‐constant of IVR) and E‐wave derived parameters, such as peak A‐V gradient, have also been established (Chung et al. 2006). Continuity of LVP contours during the ‘isovolumic relaxation – mitral valve opening – early rapid filling’ interval and the physiology of relaxation and filling requires that the forces before and after mitral valve opening (MVO) should be continuous and therefore its model predicted analogues should be correlated. Because LVP measurement (Chung model parameters) is an ‘absolute’ pressure measurement method, whereas echocardiography (PDF parameters) can only provide ‘relative’ rather than ‘absolute’ pressure information, we expect the model predicted forces to correlate, rather than be numerically identical.

Chung and Kovács (2008) have characterized the relationship between IVR and early rapid filling. They showed that the rate of pressure decay during IVR, 1/*τ*, is related to the chamber's viscous damping/relaxation (PDF) index *c*, and also the peak atrioventricular pressure gradient *kx*_*o*_ which is also equal to the initial force of early rapid filling (*F*_*i* E‐wave_). It was also shown that there is correlation between traditional IVR (IVRT, *τ*) and early filling (DT) measures.

### Low ejection fraction, high heart rate and elevated LVEDP

All ventricles at mitral valve opening must initiate filling by being mechanical suction pumps (d*P*/d*V *< 0). Therefore, the kinematics that connects IVR to suction initiated early rapid filling remain unaltered, that is, the same equations of motion for IVR (Chung model) and the E‐wave (PDF formalism) apply for low EF, high HR and elevated LVEDP. Thus, the Chung parameters and the PDF parameters will change accordingly but the correlations between terminal force of IVR and initial rapid filling force are expected to remain essentially the same, although the magnitudes of the forces are expected to be different than the forces in the “normal” cases.

### Clinical Importance and implications

The physiologic and clinical importance of this method is that it can approximate complex physiology of IVR and suction‐initiated early rapid filling using Newton's Law – provides for a linear model of events. Furthermore, linearity assures unique parameter values in solving the ‘inverse problem’ and thereby allows (clinicians and physiologists) direct determination of lumped parameters that govern the system from direct in vivo data obtainable during routine studies.

We conclude that kinematic model‐based analysis of IVR and of the E‐wave elucidates DF mechanisms common to both. The observed in vivo relationship provides novel insight into diastole itself and the causal, mechanistic, model‐based relationship that couples IVR to early rapid filling.

## Limitations

### E‐wave selection

Although the PDF formalism is applicable to all E‐waves, the most robust analysis is achieved for E‐waves that have a clear termination and are followed by diastasis. E‐wave analysis becomes less reliable when the A‐wave merges with the E‐wave and covers more than two‐thirds of the E‐wave deceleration portion. This typically occurs at HR > 90 beats/min (Hall and Kovács 1993). In the present study, our inclusion criteria required use of datasets with clearly discernible E‐waves followed by a diastatic interval (average heart rate = 62 bpm).

### Sample size

The number of datasets (*n *=* *20) may be viewed as a minor limitation but the total number of cardiac cycles analyzed (*n *=* *308) mitigates it to an acceptable degree.

## Conclusions

We derived terminal force of IVR (*F*_*t* IVR_) from kinematic modeling of IVR (Chung model) and the initial force of early rapid filling (*F*_*i* E‐wave_) from E‐wave based kinematic modeling (PDF formalism). We utilized in vivo, human, simultaneous LVP and transmitral echocardiographic E‐wave data for validation. Our results show that terminal force of IVR and initial force of early rapid filling are closely correlated. These observed in vivo relationships provide novel, model‐based insight into physiological isovolumic relaxation mechanisms and the mechanism of early rapid filling via a link of model‐predicted force‐generating chamber properties.

## Conflict of Interest

The authors declare no conflict of interest.

## The PDF Formalism

The PDF formalism characterizes Doppler transmitral velocity profiles (E‐ and A‐waves) kinematically in analogy to the motion of a damped simple harmonic oscillator (SHO). Thus transmitral blood flow velocity is the result of a balance between elastic, inertial, and damping forces. During early rapid filling, the elastic driving force, due to the previous systolic loading of elastic elements in the ventricle (titin, elastin, the visceral pericardium, and collagen (Helms et al. 1996; Jobsis et al. 2007; Katz 1930; Mizuno et al. 2000; Robinson et al. 1986) etc.), generates both acceleration‐generating forces that encounter acceleration‐opposing inertial and resistive (damping) forces. The relative balance of these three forces is reflected in the values of the three mathematically independent model‐derived parameters: *k*,* c*, and *x*_*o*_. The SHO equation of motion for the E‐wave is given by:

(A1) $$\frac{d^{2}x}{dt^{2}}+c\frac{dx}{dt}+kx=0$$

where the terms from left to right represent the inertial, resistive, and elastic forces, per unit mass, respectively. In equation (A1) *x* is the initial displacement of oscillator spring, d*x/*d*t* is the velocity of oscillator, and d^*2*^ *x/*d*t* ^*2*^ is the acceleration of oscillator. Without loss of generality equation (A1) allow computation of the parameters per unit mass by dividing through by *m*.

This is Newton's second law and solves the “inverse problem” of diastole by providing three unique parameters, *k*,* c*, and *x*_*o*_, which specify each E‐wave contour (Kovács et al. 1997). The closed form solution of equation (A1) yields E‐wave velocity, given by:

(A2a) $$v\left(t\right)=\frac{-x_{0}\cdot k}{\omega }e^{-\alpha t}\sin\left(\omega \cdot t\right)$$

where $\omega =\frac{\sqrt{4k-c^{2}}}{2}$ and $\alpha =\frac{c}{2}$ , when 4*k *> *c* ^*2*^ (underdamped kinematic regime), *ω* is the angular frequency of SHO. The phase shift in the argument of the sinusoidal function is zero because of the initial condition (at *t* = 0) that the E‐wave starts from zero velocity. Hence,

(A2b) $$v\left(t\right)=\frac{-x_{0}\cdot k}{\beta }e^{-\alpha t}\sinh\left(\beta \cdot t\right)$$

where $\beta =\frac{\sqrt{c^{2}-4k}}{2}$ , when 4*k *< c^2^ (overdamped kinematic regime)

Using the digitized E‐wave contour as input, best‐fit, mathematically unique (*x*_*o*_, *c*,* k*) parameters are obtained for each E‐wave. The three lumped parameters *c*,* k* and *x*_*o*_ account for all the global physiologic determinants of the contour. The initial displacement of the oscillator *x*_*o*_ (cm) is the model's analogue to the velocity‐time integral (VTI) of the E‐wave (i.e., related to volumetric preload) (Kovács et al. 1997). Chamber stiffness (d*P/*d*V*) is linearly related to the spring constant *k* (g/s^2^) (Kovács et al. 1997; Lisauskas et al. 2001a), while the chamber viscoelasticity/relaxation index *c* (g/s) characterizes the resistance of the filling process (Lakshminarayan K and SJ 1993; Hall and Kovács 1994). It has been shown that PDF analysis of the Doppler E‐wave can accurately determine LV diastatic (passive) chamber stiffness (Mossahebi and Kovács 2012).

## Kinematic model of isovolumic relaxation (Chung model)

In an effort to completely characterize the wide range of physiologically observed IVPD PPP trajectories Chung et. al. proposed a general model of IVPD governed by the physically intuitive interplay of inertial, stiffness and relaxation forces. These forces determine, via Newton's law, motion of the chamber manifesting as (small) displacements (isovolumic torsion, chamber shape change) (Chung and Kovács 2008). Utilizing Laplace's law to transform displacements to pressures, Chung et al. used Newton's Law (per unit mass) to account for IVPD:

(A3) $$\frac{d^{2}P}{dt^{2}}+\frac{1}{\mu }\frac{dP}{dt}+E_{k}\left(P-P_{\infty }\right)=0$$

where *µ* is a relaxation parameter, *E*_k_ is a stiffness parameter, and *P*_∞_ is the pressure asymptote.

When the recoil (*E*_*k*_ *P*) and relaxation [(1/*μ*)d*P/*d*t*] terms numerically dominate the inertial term (d^2^ *P*/d*t* ^2^ ≈ 0) (Shmuylovich and Kovács 2006), the solution to equation (A3) reduces to the familiar monoexponential solution for IVPD with τ = 1/*μE*_*k*_. As a further benefit of the approach, note that neither the monoexponential (Weiss et al. 1976) nor the logistic parameter‐predicted pressure decay (Matsubara et al. 1995) can characterize the range of physiologically encountered IVPD (as it appears in the PPP) and the data before d*P*/d*t*_min_ (Chung and Kovács 2008).
